# Integrating transcriptomic techniques and *k*-means clustering in metabolomics to identify markers of abiotic and biotic stress in *Medicago truncatula*

**DOI:** 10.1007/s11306-018-1424-y

**Published:** 2018-09-17

**Authors:** Elizabeth Dickinson, Martin J. Rusilowicz, Michael Dickinson, Adrian J. Charlton, Ulrike Bechtold, Philip M. Mullineaux, Julie Wilson

**Affiliations:** 1 Department of Chemistry, University of York, York, YO1 5DD UK; 2 Department of Computer Science, University of York, York, YO1 5DD UK; 3 Fera Science Ltd., Sand Hutton, York, YO41 1LZ UK; 4 School of Biological Sciences, University of Essex, Colchester, CO4 3SQ UK; 5 Department of Mathematics, University of York, York, YO1 5DD UK

**Keywords:** Metabolomics, *Medicago truncatula*, Drought, Biotic stress, Clustering

## Abstract

**Introduction:**

Nitrogen-fixing legumes are invaluable crops, but are sensitive to physical and biological stresses. Whilst drought and infection from the soil-borne pathogen *Fusarium oxysporum* have been studied individually, their combined effects have not been widely investigated.

**Objectives:**

We aimed to determine the effect of combined stress using methods usually associated with transcriptomics to detect metabolic differences between treatment groups that could not be identified by more traditional means, such as principal component analysis and partial least squares discriminant analysis.

**Methods:**

Liquid chromatography-high resolution mass spectrometry data from the root and leaves of model legume *Medicago truncatula* were analysed using Gaussian Process 2-Sample Test, *k*-means cluster analysis and temporal clustering by affinity propagation.

**Results:**

Metabolic differences were detected: we identified known stress markers, including changes in concentration for sucrose and citric acid, and showed that combined stress can exacerbate the effect of drought. Changes in roots were found to be smaller than those in leaves, but differences due to *Fusarium* infection were identified. The transfer of sucrose from leaves to roots can be seen in the time series using transcriptomic techniques with the metabolomics time series. Other metabolite concentrations that change as a result of treatment include phosphoric acid, malic acid and tetrahydroxychalcone.

**Conclusions:**

Probing metabolomic data with transcriptomic tools provides new insights and could help to identify resilient plant varieties, thereby increasing future crop yield and improving food security.

**Electronic supplementary material:**

The online version of this article (10.1007/s11306-018-1424-y) contains supplementary material, which is available to authorized users.

## Introduction

Legumes are an important component of sustainable food production and leguminous plants are used throughout Europe as both a food commodity and high protein source in animal feedstock. Population increase and climate change mean that problems with crop yields rapidly need addressing. Legumes are sensitive to abiotic (chemical and physical) stresses (Charlton et al. 2008), most significantly water deficit and soil salinity, with drought currently a major factor limiting crop productivity worldwide. *Fusarium oxysporum*, a soil-borne fungus, causes widespread disease in agricultural crops (“fusarium wilt”), infecting most legumes as well as other fruit and vegetables (Rispail and Rubiales 2015). This biotic stress causes vascular vessel clogging by a combination of pathogen activities and host defence responses (Beckman 1987; Di Pietro et al. 2003), leading to characteristic wilt symptoms and rapid plant death as a result of severe water stress. Disease development is favoured by warm temperatures and drought stress has been shown to enhance the proliferation and spread of *Fusarium* pathogens in cereals (Liu and Liu 2016).

*Medicago truncatula* is a small clover-like leguminous plant, that has been extremely well-studied (Young et al. 2011) and is also highly susceptible to *Fusarium* infection and drought stress (Ramírez-Suero 2010). Understanding of the changes in biochemistry due to combined *Fusarium*-drought interaction in *Medicago* could provide relevant pathway information for leguminous crops. It is well established that metabolites connect the genotype and phenotype with environmental conditions, which has recently led to the use of metabolites as selection markers in crop breeding programs (Fernie and Schauer 2009). During drought stress the, typical metabolite changes include the accumulation of carbohydrates and amino acids but also changes in phenylpropanoids leading to differential flavonoid profiles (Rasmussen et al. 2012, Bechtold et al. 2016). Similarly, metabolic profiling during fungal infection has shown a mobilization of carbohydrates, changes in amino acid pools, and the activation of isoflavonoid, *α*-linolenate, and phenylpropanoid biosynthetic pathways (Aliferis et al. 2014). However, to date little has been done to study the combined effects of biotic and abiotic stresses (Mittler and Blumwald 2010; Swindell et al. 2007), with the work reported being largely descriptive, stating how changes in humidity, salinity or temperature affect resistance to pathogens (AbuQamar et al. 2009; Bechtold et al. 2005; Yoshioka et al. 2001; Santino et al. 2013).

The use of principal component analysis (PCA) for data exploration and partial least squares (PLS) with regression or discriminant analysis for prediction and classification are standard techniques in metabolomics (Madsen et al. 2010; Hendriks et al. 2011). However, these methods are based on variance in the data and can be dominated by large variables unrelated to the problem in question (Blekherman et al. 2011). Hence subtle differences between samples or treatment groups may not be detected due to little variation between differentiating metabolites. Although less common than PCA and PLS, clustering techniques such as *k*-means and its advancements have been used successfully in metabolomic studies (Hageman et al. 2006; Li et al. 2009; Ren et al. 2015). Conversely, clustering of time-series data in transcriptomics is widely used to establish differential expression of genes and the specific time-points at which this occurs under particular perturbations to the system (Breeze et al. 2011; Heard 2011). Considering abiotic and biotic stress as perturbations on the metabolome, the application of clustering techniques and transcriptomic tools can provide similar insights from metabolomic time-course data. The integration of *techniques* from different -omics technologies provides an opportunity for integration of *data* from different technologies and a means of exploring the effects of stress from genome through to metabolome. Although new methods are emerging (Wanichthanarak et al. 2015), -omics integration is not a trivial process and the development of effective procedures is continually sought.

The Gaussian Process Two-Sample (GP2S) Test of differential expression is used in transcriptomics to test for differential gene expression between two treatment groups, by fitting an expression trend over time for each group and identifying deviations between trends (Stegle et al. 2010). This technique accounts for measurements from multiple replicates, is robust to outliers and can be used to identify differential behaviour in subintervals of the time series. The method uses a Gaussian Process to produce a “smooth” function describing the times series, created over all samples from all groups (irrespective of treatment), assuming samples are drawn from a “shared” distribution. Similarly, an alternative model describes the time series for two individual treatment groups as samples from “independent” distributions. The “shared” and “independent” models are then compared using the logarithm of the Bayes factor (“score”). The higher the log Bayes factor, the more evidence there is for the gene being differentially expressed. The scores above a threshold are used to identify genes with differential expression, therefore GP2S can act as a “filter” to remove those that are not expressed. The approach can be applied to metabolite concentrations over time, for example by filtering out changes due to growth and retaining information for only those metabolites with time series differing between treatment groups.

Developed as a preliminary step in gene regulatory network building, temporal clustering by affinity propagation (TCAP) takes account of time delays, inversions and transient correlations under specific perturbations, (Kiddle et al. 2010). An information-rich distance measure and a clustering algorithm which evaluates each data point from a data set as part of possible cluster centre, or “exemplar” are combined in an iterative procedure, where messages are exchanged between data points until a high-quality set of exemplars and corresponding clusters emerges (Frey and Dueck 2007). This technique can be utilised with metabolite concentrations to establish temporal changes due to simultaneous abiotic and biotic stress, for example, to show how stress can cause an increase in a metabolite’s concentration in the plant with a corresponding decrease in concentration of another metabolite, by clustering both time series together.

A summary of the advantages and disadvantages of these techniques are summarised in Table 1. The integration of the techniques with metabolomics will be a basis of our future investigations and long term goal of combining data from transcriptomics and metabolomics in similar studies.

**Table 1:** Summary of the advantages and disadvantages of transcriptomic techniques GP2S and TCAP

	GP2S (Stegle at al. 2010)	TCAP (Kiddle et al. 2010)
Advantages	Effectively acts as a filter to remove genes/metabolites which do not change in expression /concentration over time Computationally fast Developed to be robust to outliers Uses data from all biological replicates (not mean) to produce models Synchronized observation times are not required Can be extended to elucidate the time at which differential expression/metabolite concentration differences begin to occur	Information–rich similarity measure and clustering algorithm finds gene expression / metabolite concentrations which follow the same time series or incorporates temporal changes: a) Delays—a similar time series profile but with a lag b) Transient correlations—similar time series for some time points, not all c) Inversions—same time series profile but inverted d) Combination of the above Robust, with little user input Output simple to interpret
Disadvantages	Only compares two treatment groups at a time	Mean time series of replicates required Whilst faster than other transcriptomics clustering algorithms, it is computationally intensive

## Materials and methods

### Sample preparation and data acquisition

All the plants were presymtomatic of drought or disease stress although infection and drought conditions were confirmed by plate testing for *Fusarium oxysporum* and by monitoring pot weight and physiological response. Full details of plant growth conditions, sample collection and preparation, data acquisition and pre-processing can be found in Rusilowicz et al. 2016, but will be briefly described for clarity. For each of the four treatment groups (control (C), drought (D), *Fusarium*-infected (F) and combined stress (FD)), three plants (biological replicates) were harvested from each experimental group at daily intervals for 13 days. Each plant was removed carefully from its substrate to minimise damage to roots, shaken and the roots gently washed. Roots were dried before both leaves (L) and roots (R) were cut directly into beakers of liquid nitrogen. Only mature leaves were cut whilst dead or young leaves were discarded. After freezing, both leaves and roots were freeze dried for approximately 48 h. Lyophilised samples were then stored at room temperature.

Triplicate root and leaf samples were taken for each treatment/time point. Samples were extracted by taking 5 mg of ground freeze dried material into 1 ml of methanol:water (1:1) and shaken for 30 min. Extracts were then centrifuged, with the supernatant diluted fourfold using methanol:water (1:1) before analysis by liquid chromatography–high resolution mass spectrometry (LC–HRMS). Data was acquired in a random sample order with a quality control (QC) sample every six samples. QCs were sourced from a homogenised mixture of control samples collected from a previous similar pilot drought study of *Medicago* following a similar design (Ruscilowicz et al. 2016), as the amount of material available from experimental samples in this combined stress study was low. Both positive (+) and negative (−) mode LC–HRMS data were acquired for leaf (L) and root (R) samples, producing four datasets (L+, L−, R+, R−). Data alignment and peak picking was performed using Progenesis QI (Nonlinear Dynamics, Waters Corporation, Newcastle Upon Tyne, UK). Table 2 shows the number of peaks obtained together with the number of samples in each case.

**Table 2:** The number of samples (observations) in the data sets analysed with the number of peaks (variables) found in each

	Leaf	Root
No of samples	184 (149 + 35 QC)	182 (148 + 34 QC)
Negative mode peaks	1239	4292
Positive mode peaks	1681	4813

All observations and variables were used in each principal component analysis for each data set respectively

### Initial exploratory data analysis and pre-processing

**Fig. 1:**
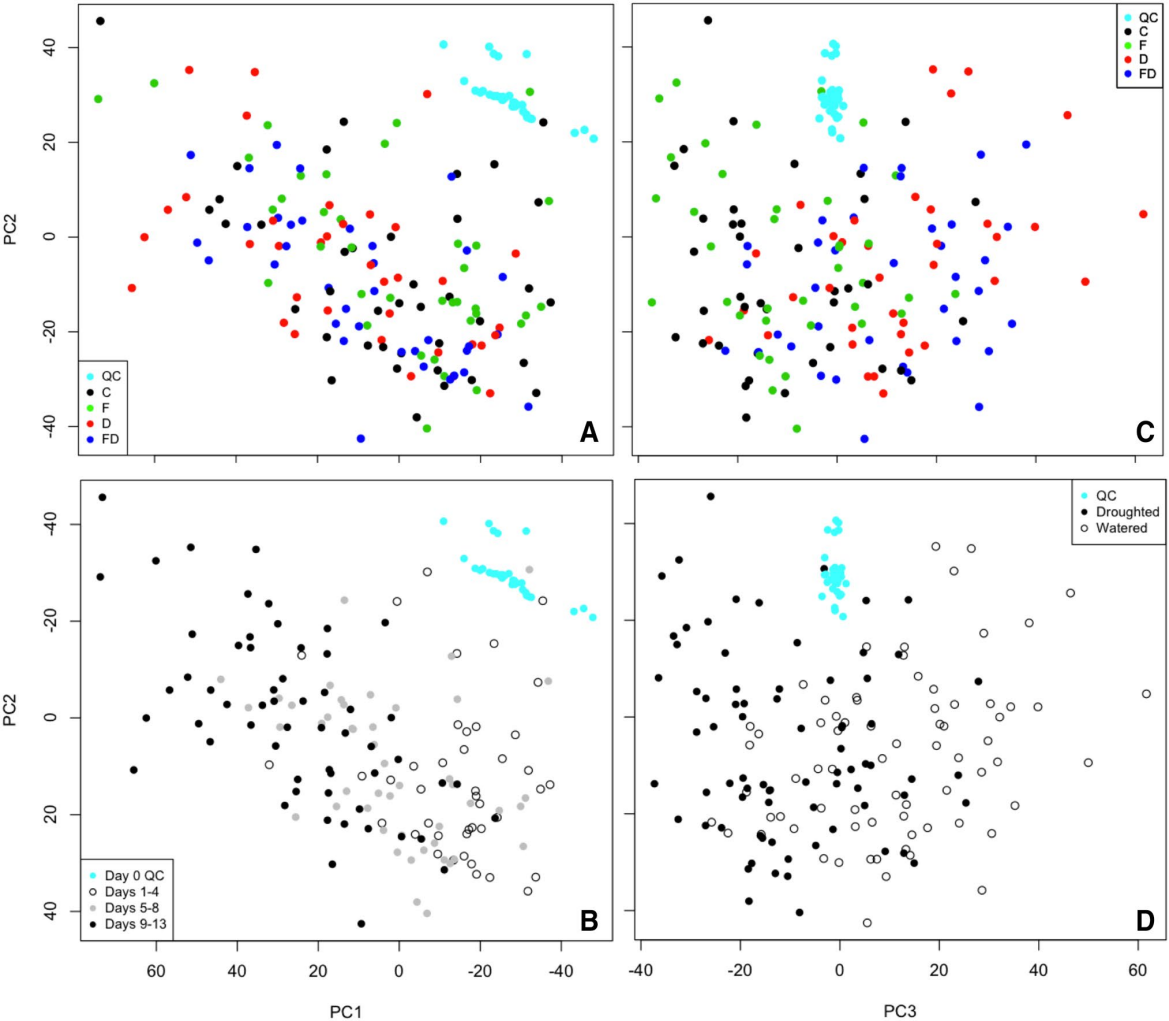
PCA scores plots for all root samples in LC–MS negative mode (R−). **a** No distinction observed between treatment groups along PC1 and PC2. **b** The greatest variance is due to plant growth. **c** No distinction is observed between C and F or between D and FD along PC3. **d** The variation along PC3 is due to differences between watered and droughted samples, though with some overlap

Analyses were conducted in R version 3.4.4 (R Core Team 2016, R Foundation for Statistical Computing, Vienna, Austria) using code written in-house. In initial data exploration PCA (dimensions of data shown in Table 2) showed batch differences to be the greatest source of variance for each dataset (Supplementary Figs. 1 and 2). To remove this technical variation, batch correction techniques were applied (Rusilowicz et al. 2016). The effectiveness of batch correction was assessed using the Bhattacharrya distance (Supplementary Table 1) (Wehrens et al. 2016). Figure 1 shows PCA scores plots obtained after batch correction and scaling. UV-scaled data were used throughout the analyses, but unscaled data were used to check for noise peaks in the final output lists. PLS-DA was also performed on the batch corrected data using tenfold cross-validation (Supplementary Fig. 3).

Filtering of the time series was achieved in two ways. GP2S (Stegle et al. 2010) was conducted via the Discovery Environment at http://www.cyverse.org (Goff et al. 2011; Merchant et al. 2016) using all three biological replicates. Recommended starting values were used for the hyperparameters of the Gaussian Processes (0.5 length scale, 1 process variance and 0.4 noise variance) and log Bayes factor score (3.0). Time series profiles for each peak were obtained from the median intensity of the three replicates at each time point (days 1–13) and the method provided a list of metabolites that differed in concentration over time for each pair of treatment groups (C + D, C + F, C + FD, D + F, D + FD, and F + FD) in the L-dataset.

In a second approach, focussed on the identification of differences related to *Fusarium* infection, the median time profiles were calculated before filtering to remove those that changed very little over time. This method was applied to the pairs of treatment groups C + F and D + FD and was achieved by applying a threshold on the profiles’ variance across the time-series and discarding profiles with low variance. As with much of cluster analysis, this step was somewhat subjective with the threshold chosen to reduce the number of time-series profiles by approximately half. Control correction was applied for the D + FD analysis to eliminate metabolic changes due to plant growth. This was achieved by subtracting the corresponding median control from each time point in the other treatment groups.

### Cluster analysis

**Fig. 2:**
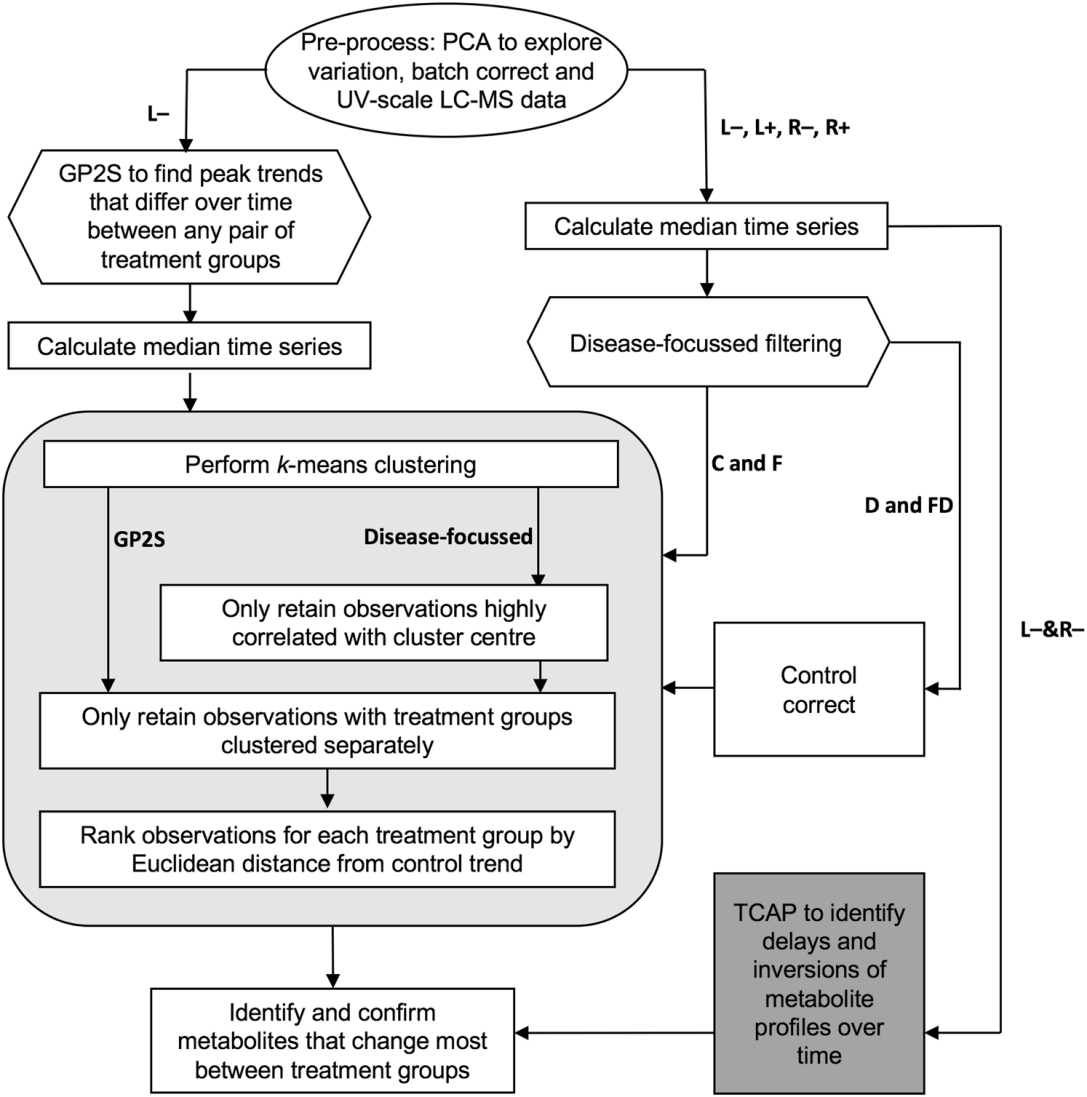
Flow diagram showing the procedure followed for pre-processing, filtering and clustering the data. For the data sets used, L and R indicate leaf and root while + and − indicate positive and negative mode data respectively

**Fig. 3:**
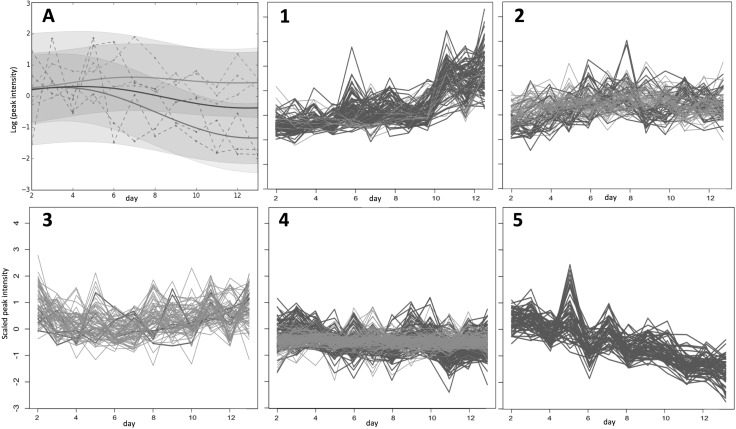
GP2S analysis of groups D (droughted, shown in green) and F (Fusarium infected, shown in red). **a** Example of GP2S output showing a deviation in trends for the peak with m/z 191.0204 at 3.61 min. The overall Gaussian fit for both groups is shown in blue. (1)–(5): Clusters obtained by k-means cluster analysis of time series passing the threshold in GP2S. The time series shown in **a** is found in cluster (3) for group F (red) and cluster (5) for group D (green). In fact, all time series in cluster (5) are from group D (green) and almost all time series in cluster (3) are from group F (red). Cluster (1) also shows quite a strong association with treatment group D (mostly green)

**Fig. 4:**
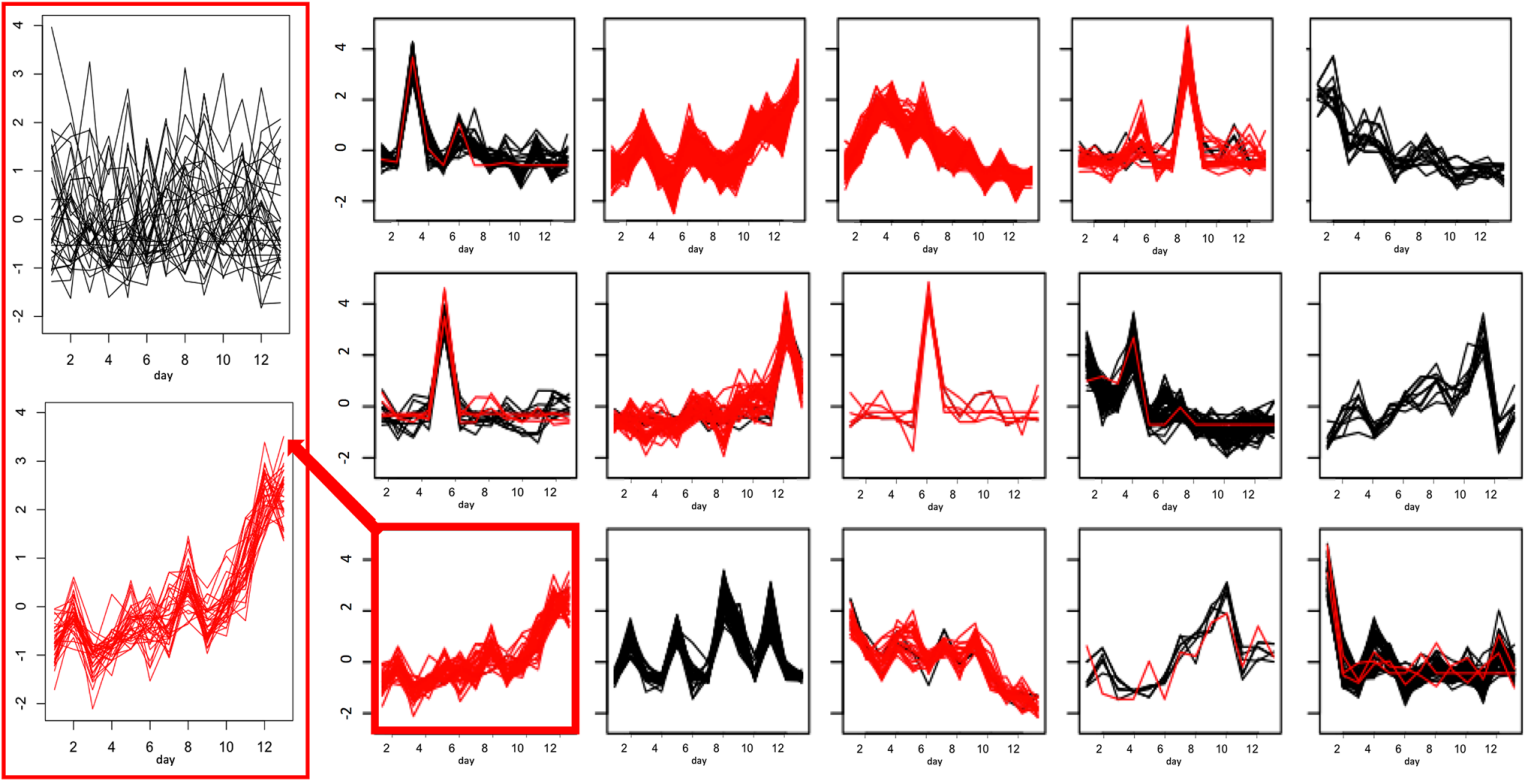
Results of k-means cluster analysis of time series for groups C (control, black) and Fusarium infected (F, red) for negative mode root (R-). The x-axis corresponds to time and the y-axis to peak intensity. Cluster 11 (highlighted) contains all sucrose peaks for group F and is expanded on the left with the corresponding peaks for group C above showing no overall trend

*k*-means clustering of the time-series profiles, using the Hartigan–Wong algorithm was performed in R following the schematic shown in Fig. 2. The Elbow method (Charrad et al. 2014) was used to determine the number of clusters, giving *k* = 5 for the GP2S filtered data (Fig. 3) and *k* = 15 for the disease-focussed approach. In both cases, further filtering of the clustered time-series was performed by first removing those that had a correlation coefficient < 0.9 with the cluster centre. An example of the resulting focussed clusters is shown in Fig. 4. These more highly-correlated observations were then reduced further by removing any for which the two profiles corresponding to the same peak (one from each group) clustered together. The remaining time-series, representing peaks that differ between the two groups being analysed, were then ranked to determine the profiles that changed most over time as a result of drought, *Fusarium* infection or combined stress. For each peak represented, the Euclidean distance between the time series for each treatment group (D, F, and FD) and the control group (C) was calculated from the original batch-corrected data and used to rank the peaks. For the highest ranked peaks, F-tests were performed to compare the variance within the controls with the variance in each treatment group (Table 3). For these tests the data were batch corrected, but not scaled, and the median control value for the appropriate day was subtracted from each observation, including the controls to remove any growth effects.

**Table 3:** Confirmed metabolites (identified to level 1) with time series that clustered according to treatment group

Data set	METLIN ID	Metabolite [most abundant molecular species]	Observation		Significance in F-test
			*m*/*z*	RT (m)	
L–	137	Sucrose [M–H]^−^	341.1079	2.15	D ***; F *; FD ***
	124	Citrate [M − H]^−^	191.0203	2.47	D **; FD **
	3328	Isocitrate [M − H]^−^	191.0203	3.61	D **; FD **
	118	Malic acid [M − H]^−^	133.0147	2.28	D *; FD **
L+	137	Sucrose [M + Na]^+^	365.1053	2.15	D ***; FD ***
	124	Citrate [M + Na] ^+^	215.0162	2.61	D ***; FD ***
	3328	Isocitrate [M + Na] ^+^	215.0162	3.37	D ***; FD ***
	45,732	*Tetrahydroxychalcone	273.0757	17.01	F*
R–	137	Sucrose [M − H]^−^	341.1079	2.30	F^†^
	124	Citrate [M − H]^−^	191.0193	3.45	FD^†^
	3328	Isocitrate [M − H]^−^	191.0193	4.03	D *; F *; FD*
R+	3231	Phosphoric acid [M + H]^+^	98.9844	2.05	D^†^

Significance levels for the F tests comparing the variance of treatment groups with that of the controls are indicated by: ***< 0.001; **< 0.01; *< 0.05; ^†^< 0.1 (*tetrahydroxychalcone was only identified to level 2)

### Temporal clustering by affinity propagation (TCAP)

To reduce computation time, the median time-series profiles for the L- and R-datasets were filtered as described above to remove those with low variance. The resulting profiles from the two datasets were submitted together for analysis by TCAP version 2 (Kiddle et al. 2010) conducted in Matlab R2105a (The Mathworks, Inc, Natick, MA, USA) with the self-similarity input set at − 8.

## Results

After batch correction, PCA showed that most variance was due to plant growth (Fig. 1). Whilst variation between watered and droughted plants can be seen along PC3 with overlap between early day observations, no separation is observed between D and FD, or between C and F (i.e. *Fusarium* effects). This is also the case for PCA conducted with only C and F groups or D and FD groups included to remove the dominance of drought (Supplementary Fig. 4). Furthermore, PLS-DA did not highlight any disease-related differences (Supplementary Fig. 3).

Using the GP2S approach, the log Bayes scores revealed differences between the pairs of treatment groups, C + D, C + FD, F + D and F + FD, i.e. drought effects, but no differences between the profiles for D + FD and C + F, i.e. *Fusarium* effects, were detected above the threshold set. Interestingly, more differences above the threshold were detected when the analyses involved the combined stress group, with 332 and 398 peaks highlighted for C + FD and F + FD respectively, in comparison to 226 and 250 peaks for the analyses involving drought, C + D and F + D respectively. Figure 3 shows the *k*-means clustering of the time series for metabolites passing the threshold in the F + D analysis with three of the five clusters showing a very strong relationship with one particular treatment group, showing that certain trends are associated with a particular group. This separate clustering of treatment groups also occurred for the C + D, C + FD and F + FD GP2S analyses.

As the GP2S approach did not reveal differences between the groups D and FD or C and F, a different approach to filtering was adopted with these groups in order to identify disease-related differences. Figure 4 shows the results of k-means clustering of the C and F data, using the alternative method. The time series are highly correlated with cluster centres and show clustering according to group as seen for profiles identified by GP2S. As the observations for which the time-series for both groups occurred in the same cluster have been removed, the time-series for any peak represented here must show a different trend in the two groups. The close-up of Cluster 11 in Fig. 4, for example, contains observations from the F group only with the corresponding “partner” observations for the C group either appearing in a separate cluster or having been filtered out due to little change over time.

When all four treatment groups were considered, drought-related responses dominated both leaf and root analyses for both LC-MS modes with time series for watered plants (C/F) clustering separately from droughted observations. The Euclidean distance was calculated using unscaled data to identify the time series that differed most from the controls. Table 3 shows the highest ranked metabolites with confirmed identifications (level 1 as described in Salek et al. 2013) with the significance levels obtained for F tests between each treatment group and the control group. For many of the highly-ranked metabolites the changes only appear to be significant in the droughted groups (D and FD), however, sucrose and citrate also differ significantly with *Fusarium* infection (F), with this group presenting the only significant changes in some cases. Furthermore, changes in tetrahydroxychalcone were only significant for the diseased group (F).

**Fig. 5:**
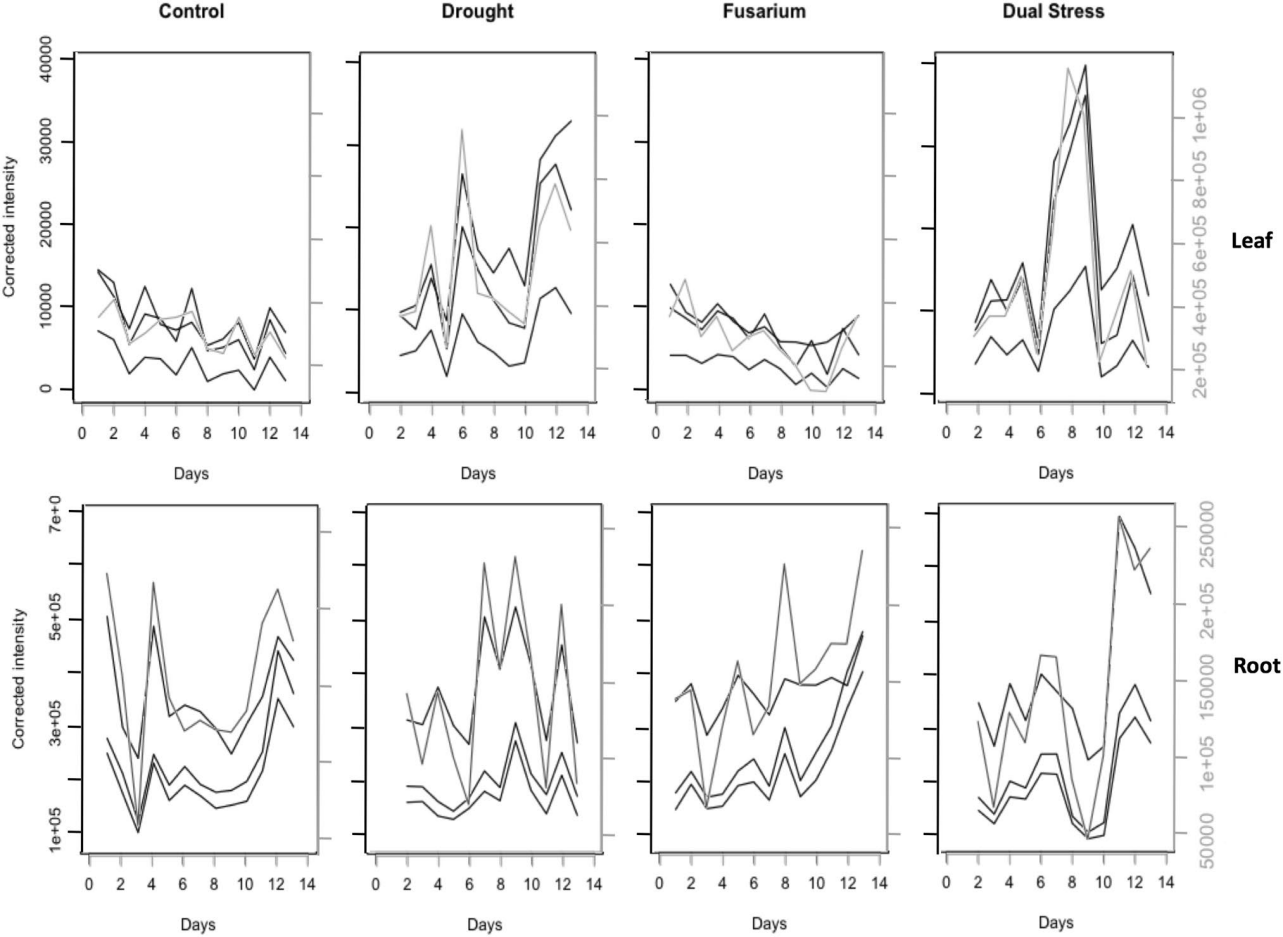
Time series profiles for sucrose for each treatment group. Both positive (grey) and negative (black) ion modes show the same overall trend. Leaf and root clearly exhibit different response to drought and dual stress

Further metabolites, either confirmed or affirmed (level 2 identification) with time series showing different trends to the controls are given in Supplementary Tables 2–5. Standards confirmed sucrose and citrate/isocitrate to be the metabolites responding most to drought stress, in line with previous observations on *Arabidopsis thaliana* and *Thelungiella salsuginea* (Pinheiro et al. 2018; Bechtold et al. 2016). However, the disease focussed approach, using only C and F data or only D and FD data, showed the trends for sucrose and citrate peaks, as well as malic acid, to be different for diseased (F or FD) and non-diseased (C or D) groups. Figure 5 shows the time series for sucrose peaks with both positive and negative LC-MS modes highlighting differences between treatment groups for both leaf and root. Whilst drought-related differences dominate in leaf, with observations from watered groups, C and F, showing the same slight downward trend, there is a consistent difference between droughted (D) and combined stress (FD) observations, with an increase in late stress for D not seen for FD. In roots, watered groups show a rise in late stress, with a final decrease seen for C but not F and the times-series profiles differ greatly between drought and combined stress. The highest sucrose concentration in D occurs between days 6–9, but not until days 11–13 in FD. In D, this increase in root corresponds to a decrease in leaf, whereas in FD a dip before the major increase in sucrose concentration in root corresponds to the highest levels in leaf. Citrate levels decrease with drought (D and FD) and present large fluctuations that occur on alternating days between leaf and root in the combined stress group (Supplementary Fig. 5).

**Fig. 6:**
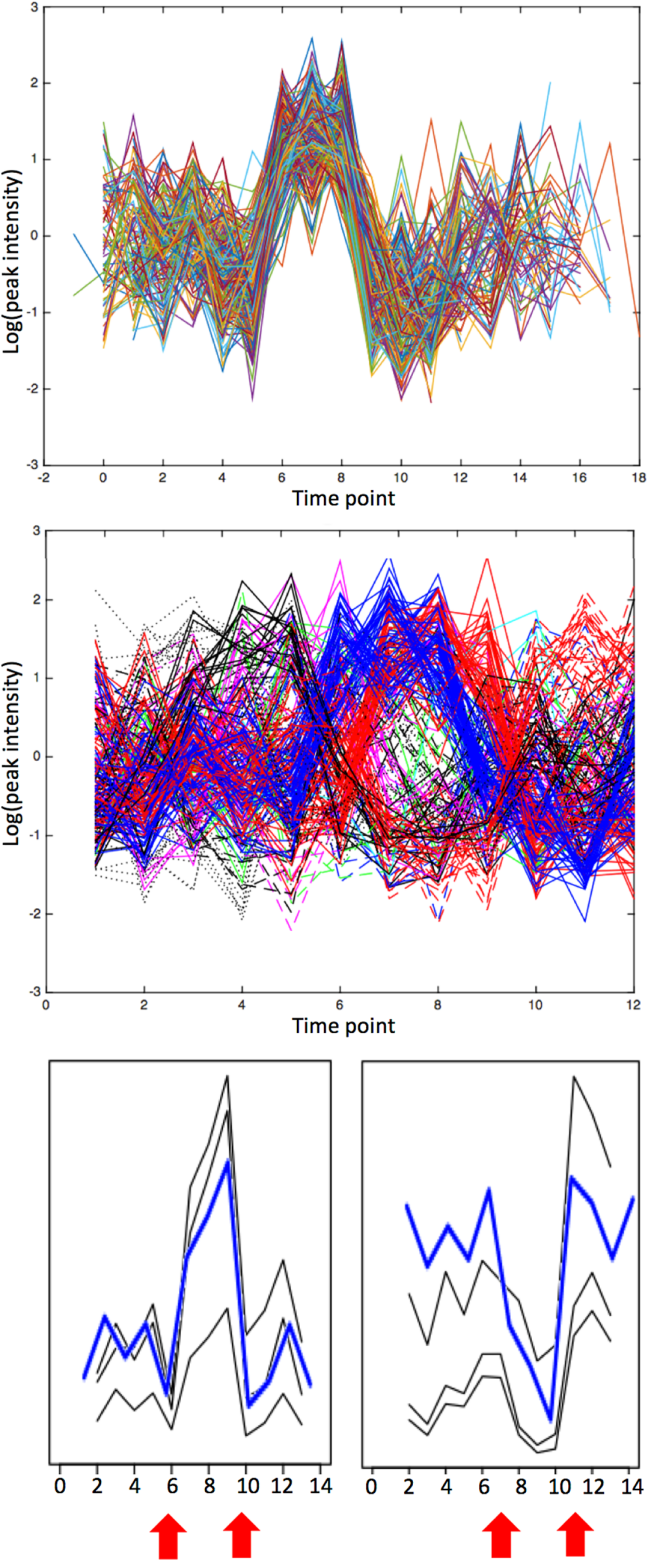
Cluster 40 from TCAP analysis, with time series for sucrose from both leaf and root from the dual stress (FD) group clustered together. Top: time series for all peaks within the cluster, shifted to reveal the general profile. Middle: time series coloured according to unshifted patterns. Bottom: sucrose time series from group FD for root (right) inverted and delayed by 1 day from leaf (left). The red arrows indicate the start and end days of the major concentration increase/decrease

To investigate the use of temporal clustering by affinity propagation (TCAP) with metabolomics data, the negative mode datasets for leaf and root were combined after preliminary filtering. We found that citrate peaks for different treatment groups did not cluster together and that leaf and root time series occurred in separate clusters. This was also true of sucrose for three of the four treatment groups (C, F and D), but allowing delays and inversions to be considered in the cluster analysis led to sucrose peaks for both root and leaf appearing in the same cluster in the combined stress group (FD). The peak intensity profile over time obtained for sucrose in root FD is an inversion with a delay of one day from the leaf FD profile i.e. the sucrose concentration in leaf is maximal between days 7–9 and falls at day 10, while in root, it is at a minimal for days 8–10 and increases at day 11 (Fig. 6). The incorporation of time delays and inversions is clearly applicable in metabolomics as well as in transcriptomics.

## Discussion and conclusions

Drought stress in leguminous plants primarily affects photosynthesis and cell growth (Araújo et al. 2015) with carbon metabolism affecting the concentration of sugars, used as secondary intracellular signalling messengers (Chaves et al. 2009). These soluble sugars such as sucrose interact with plant hormones under plant stress and previously have been found to increase in concentration as a result of drought with an accumulation in the leaf as photosynthesis continues despite a deficit of water (Chaves 1991). This explains the trends observed in our leaf data sets for droughted plants (D and DF) in the leaf data sets. However, a decrease in soluble sugars has been observed previously for severe drought stress (Pinheiro et al. 2001), which could explain the late decrease in sucrose concentration in leaf. Whilst F-tests show that both droughted groups have similar significance for the difference in variance from the controls, the decrease in sucrose concentration occurs earlier in the FD group (day 10) than in the D group (days 12–13), suggesting that combined stress exacerbates the effect of drought, and the combined effect of *Fusarium* infection and drought lead to a more urgent stress response. As *Fusarium oxysporum* causes vascular blockage and dehydration of the plant (Di Pietro et al. 2003), it would be expected that metabolic changes due to infection resemble those for drought.

Our results support earlier findings (Sanchez et al. 2008), which suggest that model legumes suffering from abiotic stress show an increase in osmoprotectors such as sugars, with a concurrent decrease in many organic acids such as citric acid and malic acid. It has been suggested that this depletion of organic acids is due to the reallocation of fixed carbon to the much-needed synthesis of sucrose from photosynthesis. Changes in concentration found for dehydroascorbate, the oxidised form of antioxidant ascorbic acid, in both leaf and root particularly in treatment groups D and FD, agrees with other investigations in the literature (Potters et al. 2004). Dehydroascorbate is involved in glutathione metabolism, another well-known antioxidant, therefore the related increase in the oxidised form glutathione disulphide in treatment groups D and FD again provides evidence of oxidative stress. An increase in flavonoid secondary metabolites provides evidence of plant response to pathogen infection (Falcone Ferreyra et al. 2012). Similarly, the accumulation of amino acids due to stress-induced protein breakdown, the down regulation of energy consuming processes and shift from growth to survival are all observed for the treatment groups, particularly in those affected by drought (Witt et al. 2012; Muscolo et al. 2015).

It is clear from Table 3 that changes are more extreme in leaf than in root and that significant differences in leaf are dominated by drought response, although tetrahydroxychalcone (butein) shows significant changes only with disease (F). This chalcone is a precursor to flavonoid synthesis in leguminous plants (Mierziak et al. 2014). Flavonoids are known to have antimicrobial effects, including potent antifungal activity against *Fusarium oxysporum* (Galeotti et al. 2008). Only one sucrose species shows a significant difference between the variance in the controls and that in the diseased group (F), but changes in malic acid concentration due to combined stress are an order of magnitude more significant than for drought. The pathogen infects the root (Di Pietro et al. 2001) and, although changes are considerably smaller in root than in leaf, it is noticeable that there are more significant changes due to *Fusarium* infection and combined stress in root. An increase in phosphoric acid concentration seen for control and *Fusarium*-infected plants shows that the beneficial root-associated mycorrhizal fungi are functioning normally in modulating the plant metabolome, improving water uptake and nutrition by elevating phosphate supply (Schweiger et al. 2014). In drought conditions, this modulation is adversely affected and phosphates cannot be taken up by the plant, explaining the relatively unchanged levels of phosphoric acid over time for D/FD groups.

The export of sugars from wilting leaf to growing roots is necessary to ensure plant growth (Chaves 1991), and the fall in sucrose concentration exhibited in the leaf FD at day 10 could explain the corresponding rise in sucrose in root FD, identified by TCAP analysis. Although computationally intensive, this tool developed for transcriptomics has proved useful in metabolomics. We suggest using this technique as a final step after filtering to reduce the number of time series, as demonstrated here.

The use of GP2S allowed identification of metabolic changes due to drought, but not *Fusarium* infection, whereas the disease focussed approach allowed more subtle differences to be found (i.e. between D and FD or between C and F). Our investigations showed that the suggested threshold used in GPS2 was too high for such differences. However, lowering the threshold did not improve the results and the use of GPS2 as an alternative first filter missed metabolites that were identified using the filter based on variance. Furthermore, clustering could suggest metabolites are involved in the same biochemical pathway or process for example, citrate and malic acid, whereas GP2S only identifies information on plant response other than deviating time series trends that deviate between treatment groups. We therefore conclude that the focussed *k*-means procedure, which filters out time-series based on variance, then removes those for which different treatment groups cluster together before finally ranking according to difference from the control is the most appropriate.

The probing of metabolomic data using transcriptomic techniques and new clustering procedures has proven to be effective in identifying changes in metabolite concentrations and biological processes of plants subjected to simultaneous abiotic and biotic stress. The focussed *k*-means clustering has been effective in identifying differences that were not revealed using the standard chemometric techniques of PCA and PLS-DA. The method was also more effective than the GPS2 method used in transcriptomics and, in addition to expected stress responses confirming the results of other investigations in the literature, has identified more subtle changes, providing insight into the mechanism of combined abiotic and biotic stress (combined stress) on leguminous plants that could aid the identification of more resilient varieties to improve crop yield. The TCAP analysis, also borrowed from transcriptomics did prove useful however, showing that delays and inversions can provide additional information and should be considered in the analysis of metabolomics time course data.

## Electronic supplementary material

Below is the link to the electronic supplementary material.

Supplementary material 1 (DOCX 1620 KB)
