# Supplementary material for: Integrating transcriptomic techniques and k-means clustering in metabolomics to identify markers of abiotic and biotic stress in Medicago truncatula
Source: Metabolomics. 2018 Sep 17;14(10):126. doi: 10.1007/s11306-018-1424-y (PMC6153691; doi:10.1007/s11306-018-1424-y)
Supplement: Supplementary file 1 — Supplementary material 1 (DOCX 1620 KB) [file 11306_2018_1424_MOESM1_ESM.docx]

**Supplementary Tables**

**Supplementary Table 1**: Mean Bhattacharrya distance between PCA scores for different LC-MS batches, before and after removal of batch differences. (*Figure in brackets excludes batch 7, the most different from all other batches. See Supplementary Figures for scores plots before and after correction.)

| **Data Set** | **Before batch correction** | **After batch correction** |
| --- | --- | --- |
| Root negative mode (R–) | 1.887 | 0.146 |
| Root positive mode (R+) | 5.520 | 0.060 |
| Leaf negative mode* (L–) | 33.107 (1.423)* | 0.043 (0.044)* |
| Leaf positive mode (L+) | 1.277 | 0.064 |

**Supplementary Table 2**: Confirmed metabolites (identified to level 1) changing in concentration in leaf as a result of *Fusarium* infection, drought or dual stress.

| **METLIN ID** | **Compound** | ***m/z*** | **RT** | **Pattern over time** | **Suggested biochemical impact** |
| --- | --- | --- | --- | --- | --- |
| 118 | Malic acid | 133.0148 | 2.3 | D and FD decrease | Energy metabolism |
| 137 | Sucrose | 341.1089 | 2.2 | D and FD increase | Energy metabolism |
| 124 | Citrate | 215.0163 | 3.6 | D and FD decrease | Energy metabolism |
| 63191 | Dehydroascorbate | 175.0237 | 3.4 | D and FD decrease | Glutathione metabolism |
| 3231 | Phosphoric acid | 98.9844 | 2.1 | D and FD decrease | Oxidative phosphorylation |
| 15 | Aspartic acid | 134.0448 | 2.0 | D and FD decrease | Fatty acid oxidation |
| 25 | Lysine | 147.1128 | 1.9 | FD only increase | Lysine biosynthesis |
| 13 | Arginine | 175.1190 | 1.9 | C only increase | Arginine and proline metabolism |
| 136 | Mannose | 203.0526 | 2.1 | C only increase | Energy metabolism |

**Supplementary Table 3**: Affirmed metabolites (identified to level 2) changing in concentration in leaf as a result of *Fusarium* infection, drought or dual stress.

| **METLIN ID** | **Compound** | ***m/z*** | **RT** | **Pattern over time** | **Suggested biochemical impact** |
| --- | --- | --- | --- | --- | --- |
| 35473 | Threonic acid | 135.0305 | 2.1 | D and FD decrease | Glutathione metabolism |
| 66361 | Dehydro-D-arabinono-1,4-lactone | 147.0288 | 3.4 | D and FD decrease | Glutathione metabolism |
| 44 | Glutathione Disulfide | 307.0836 | 4.2 | D and FD increase | Glutathione metabolism |
| 48214 | Pisatin | 315.0863 | 14.9 | D, F and FD increase | Flavonoid metabolism |
| 3399 | Pelargonidin | 269.0459 | 15.1 | FD only increase | Flavonoid metabolism |
| 93879 | Medicagenic acid 3-O-b-D-glucuronide ester | 543.2436 | 13.9 | F and FD decrease | Alkaloid metabolism |
| 5846 | Undecanedioic acid | 215.1296 | 14.7 | FD only increase | Fatty acid metabolism |
| 3282 | Acetyl-homoserine | 184.0581 | 2.4 | D and FD decrease | Cysteine and methionine metabolism |
| 41154 | Gentiopicrin | 379.0999 | 12.0 | D and FD increase | Alkaloid metabolism |
| 66869 | Deoxyinositol | 187.0578 | 2.4 | D and FD increase | Energy metabolism |
| 4156 | Ferulic acid | 195.0653 | 12.0 | D and FD increase | Phenylpropanoid synthesis |
| 95685 | Salicylic acid glucoside | 323.0739 | 11.5 | F only increase | Plant hormone transduction |
| 28 | Phenylalanine | 166.0863 | 10.0 | FD only increase | Tryptophan metabolism |
| 3344 | Sedoheptulose | 209.0672 | 2.2 | D and FD increase | Carbon fixation |
| 4080 | 4-Methylumbelliferone glucuronide | 353.0867 | 13.7 | F only increase | Unknown |
| 45732 | Tetrahydroxychalcone (Butein) | 273.0757 | 17.0 | D and FD increase | Flavanoid metabolism |

**Supplementary Table 4**: Confirmed metabolites (identified to level 1) changing in concentration in root as a result of *Fusarium* infection, drought or dual stress.

| **METLIN ID** | **Compound** | ***m/z*** | **RT** | **Pattern over time** | **Suggested biochemical impact** |
| --- | --- | --- | --- | --- | --- |
| 124 | Citrate | 215.0163 | 3.6 | D and FD decrease | Energy metabolism |
| 5161 | Glycerol-3-phosphate | 173.0206 | 2.0 | D and FD decrease | Energy metabolism |
| 43917 | Formononetin | 269.0806 | 15.8 | F, D and FD increase | Flavonoid metabolism |
| 63191 | Dehydroascorbate | 175.0237 | 3.4 | F, D and FD increase | Glutathione metabolism |
| 3231 | Phosphoric acid | 98.9844 | 2.1 | D and FD decrease | Oxidative phosphorylation |
| 23 | Isoleucine | 132.1019 | 4.3 | D and FD increase | Valine, leucine and isoleucine metabolism |

**Supplementary Table 5**: Affirmed metabolites (identified to level 2) changing in concentration in root as a result of *Fusarium* infection, drought or dual stress.

| **METLIN ID** | **Compound** | ***m/z*** | **RT** | **Pattern over time** | **Suggested biochemical impact** |
| --- | --- | --- | --- | --- | --- |
| 3358 | N-acetyl-galactosamine | 222.0970 | 2.2 | D and FD decrease | Energy metabolism |
| 49588 | Cirsimaritin | 315.0861 | 18.3 | F increase | Flavonoid metabolism |
| 66361 | Dehydro-D-arabinono-1,4-lactone | 147.0288 | 3.4 | F, D and FD only remain lower | Glutathione metabolism |
| 367 | 6-Phosphogluconate | 275.0175 | 2.1 | F only decrease | Energy metabolism |
| 3389 | Glucosamine 1-phosphate | 300.0490 | 2.1 | F, D and FD decrease | Energy metabolism |
| 66232 | (alpha-D-Galactosyl)-sn-Glycerol_3-phosphate | 333.0593 | 2.1 | F, D and FD decrease | Energy metabolism |
| 36000 | Octadecenedioate | 311.2228 | 18.3 | F, D and FD remain lower | Fatty acid metabolism |
| 3424 | Vernolic_acid | 295.2279 | 19.4 | F, D and FD remain lower | Fatty acid metabolism |
| 318739 | Ethyloleate | 293.2124 | 19.8 | F, D and FD increase | Lipid metabolism |
| 63171 | 2(α-D-Mannosyl)-D-glycerate | 267.0722 | 2.3 | F, D and FD only remain lower | Energy metabolism |
| 345 | Gluconic acid | 219.0473 | 2.1 | D and FD increase | Pentose phosphate metabolism |
| 364 | Hydroxy-tryptophan | 221.0919 | 10.5 | D and FD increase | Tryptophan metabolism |
| 41038 | Usnic acid | 343.0824 | 18.2 | F, D and FD increase | Aromatics metabolism |

**Supplementary Figures**

**Supplementary Figure 1**: PCA scores plots of root data obtained by negative mode LC-MS analysis (top) and positive mode (bottom), coloured by LC-MS batch. The greatest variation between samples is mostly due to batch differences (left), which are removed by QC correction (right).

**Supplementary Figure 2**: PCA scores plots of leaf data obtained by negative mode LC-MS analysis (top) and positive mode (bottom), coloured by LC-MS batch. Batch differences (shown on plots to the left) were removed (right) by QC correction for negative mode and by background correction for positive mode.

**Supplementary Figure 3**: PLS-DA scores plots for root data obtained by negative mode LC-MS analysis. Although there is separation due to drought, no pattern with infection can be observed. Using venetian blinds cross validation, 51% correct prediction was obtained when all four groups were included.


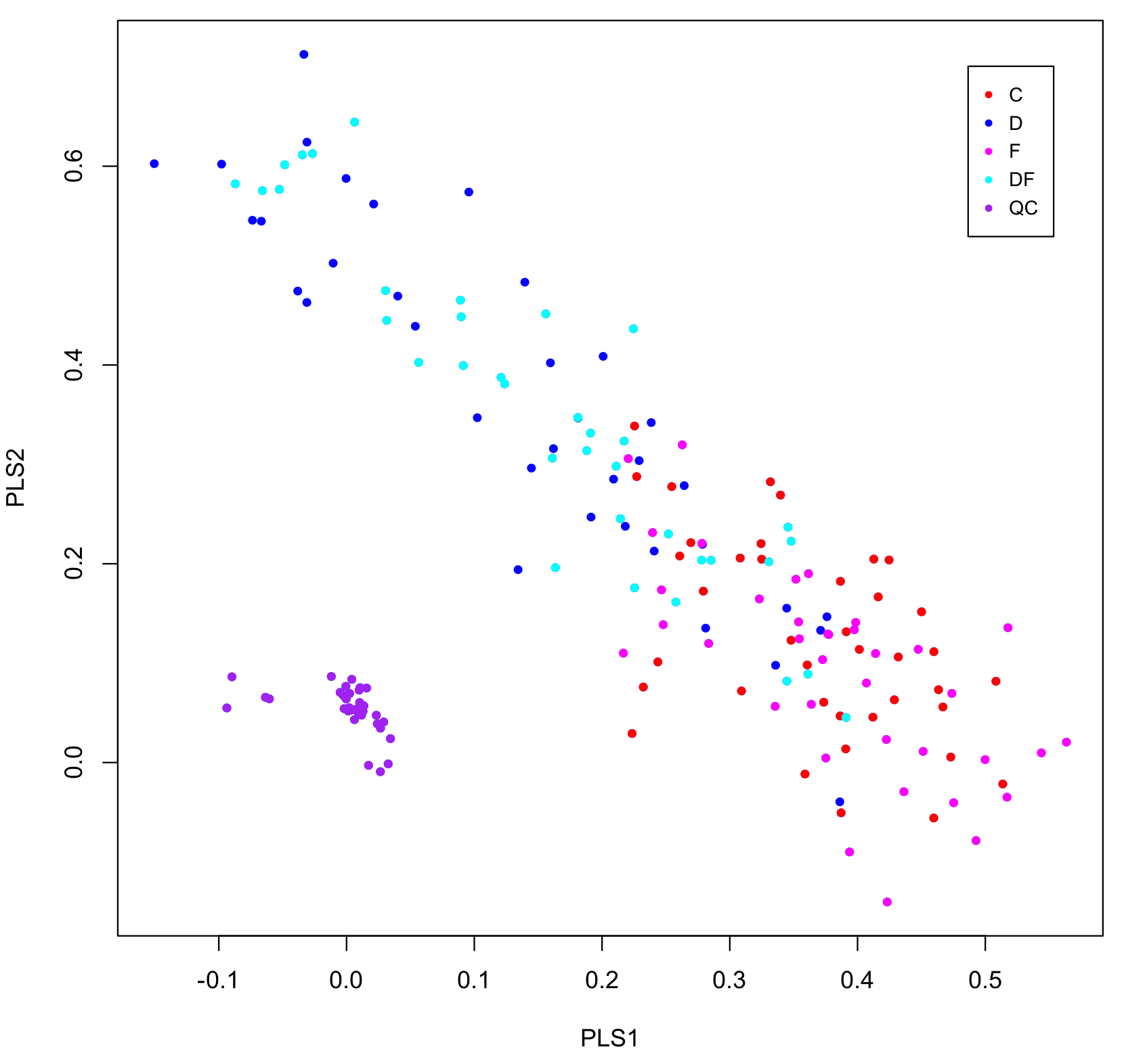


**Supplementary Figure 4**: PCA scores plots from focussed investigation of negative leaf data to investigate *Fusarium* effects; (top) C and F groups and (bottom) D and FD groups. There is no obvious pattern related to disease status.


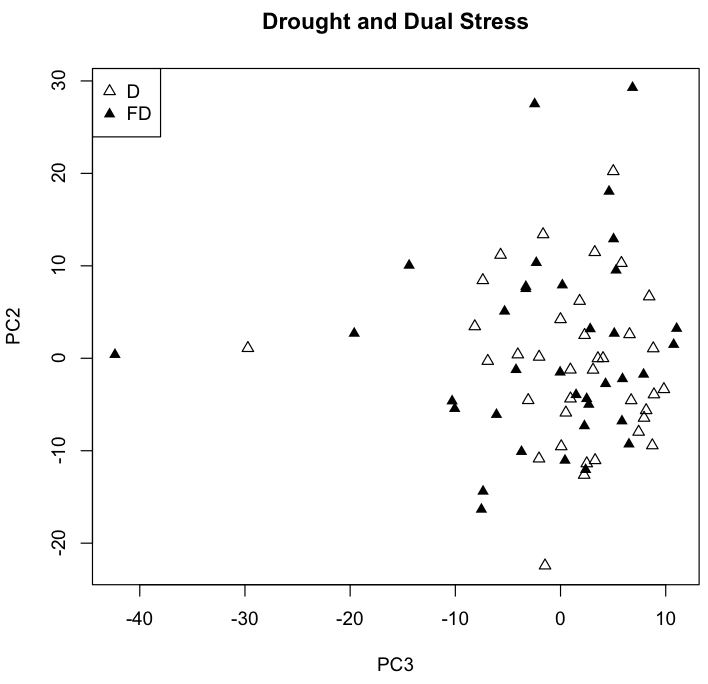

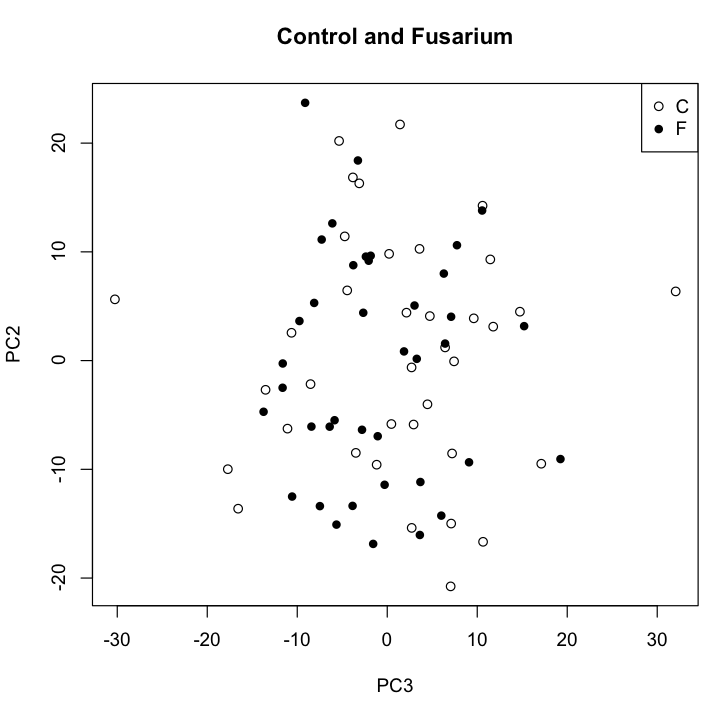

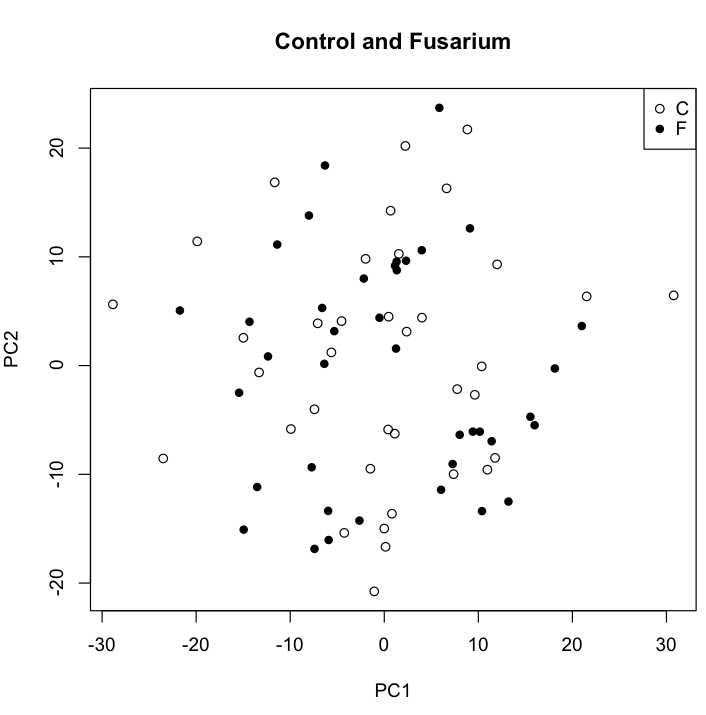

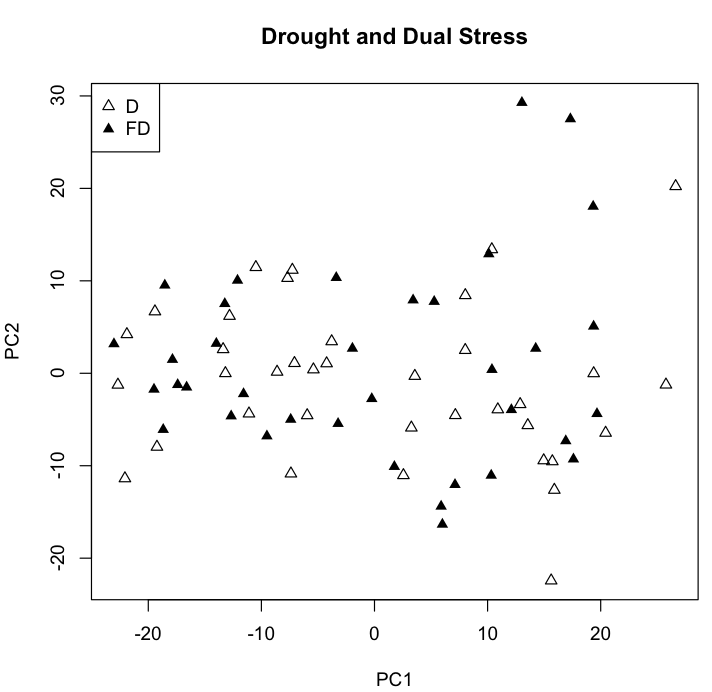


**Supplementary Figure 5**: Time series profiles for citrate/isocitrate for each treatment group. Both positive (purple) and negative (black) LC-MS modes show the same overall trend.
